# Research on the Enhancement of Laser Radar Range Image Recognition Using a Super-Resolution Algorithm

**DOI:** 10.3390/s20185185

**Published:** 2020-09-11

**Authors:** Yu Zhai, Jieyu Lei, Wenze Xia, Shaokun Han, Fei Liu, Wenhao Li

**Affiliations:** Beijing Key Lab for Precision Optoelectronic Measurement Instrument and Technology, School of Optics and Photonics, Beijing Institute of Technology, Beijing 100081, China; zhaiy_0108@163.com (Y.Z.); jieyu_lei@163.com (J.L.); xiawenze@126.com (W.X.); 00001liufei@163.com (F.L.); liwenhao_bitgd@163.com (W.L.)

**Keywords:** range image SR algorithm, target recognition, combined moment invariant, neural network

## Abstract

This work introduces a super-resolution (SR) algorithm for range images on the basis of self-guided joint filtering (SGJF), adding the range information of the range image as a coefficient of the filter to reduce the influence of the intensity image texture on the super-resolved image. A range image SR recognition system is constructed to study the effect of four SR algorithms including the SGJF algorithm on the recognition of the laser radar (ladar) range image. The effects of different model library sizes, SR algorithms, SR factors and noise conditions on the recognition are tested via experiments. Results demonstrate that all tested SR algorithms can improve the recognition rate of low-resolution (low-res) range images to varying degrees and the proposed SGJF algorithm has a very good comprehensive recognition performance. Finally, suggestions for the use of SR algorithms in actual scene recognition are proposed on the basis of the experimental results.

## 1. Introduction

With the advancement and development of technology, obtaining three-dimensional (3D) range images using laser radar (ladar) is easy and popular in recent years [1,2]. The recognition technology of the target object in the 3D range image is also extensively used in various fields such as high-altitude detection, precision guidance, automatic driving and smart homes and is a remarkable possibility for expansion remains in the future [3,4]. Owing to the limitation of hardware conditions and cost, the spatial resolution of the obtained 3D range images is typically low. Therefore, determining how to improve the spatial resolution of 3D range images through algorithms has become an important research direction of 3D imaging. This interest raises the question of whether we can improve the recognition rate of low-resolution (low-res) range images by increasing their spatial resolution.

Super-resolution (SR) of images can also be called up-sampling of images, which is a signal processing technique for estimating a high-resolution (high-res) image from a low-res image [5]. SR algorithms for range images are developed on the bases of SR algorithms for intensity images and can be classified into two categories according to the difference in input data sources. One uses only single- or multi-frame low-res range images such as bicubic interpolation (Bicubic) proposed by Hou [6], convex projections proposed by Stark [7], neighbor embedding proposed by Chang [8] and self-guided residual interpolation proposed by Konno [9]. Another uses the information of high-res intensity images of the same scene to guide the SR of range images [10] such as joint bilateral filter (JBF) proposed by Kopf [11], weighted mode filtering proposed by Min [12], Markov random field (MRF) proposed by Diebel [13] and total generalized variation (TGV) proposed by Ferstl [14]. Considering that these algorithms must strictly align high-res intensity images and low-res range images, initially registering the two images through the registration algorithm is necessary. In addition, SR methods based on deep learning have also made significant progress in recent years [15,16]. However, the current research on the role of the SR algorithm in recognition is focused on specific fields such as 2D face recognition [17,18]. To the best of our knowledge, no relevant study covers the role of the SR algorithm in range image recognition.

This work aims to propose an SR algorithm for range images based on self-guided joint filtering (SGJF). The algorithm adds the interpolation result of the range image as a guide image to the filter kernel to reduce the influence of the intensity image texture on the super-resolved image. In addition, an SR recognition system for range images is constructed and a range image acquisition platform is designed to test the effect of SR algorithms in recognizing ladar range images. Four SR algorithms are tested, including Bicubic, JBF, MRF and our proposed SGJF. The SR recognition system uses combined moment invariants composed of seven Hu and three affine moment invariants to describe the global features of the image. Moreover, it uses a back propagation neural network (BPNN) to classify and recognize the features. The recognition rates of the SR recognition system under different SR algorithms, SR factors, model library sizes and noise conditions are tested via experiments. The results show that the SGJF algorithm we proposed can largely avoid the problem of ‘texture transfer’ in the JBF algorithm and is also robust to noise. All of the SR algorithms we tested can improve the recognition rate of low-res range images and a high SR factor increases the improvement. Regardless of the resolution of the model library, high resolution of the super-resolved scene raises the recognition rate and using the model library with the same resolution as the super-resolved scene can obtain the highest recognition rate. Finally, following the experimental results, the application suggestions of SR algorithms in actual scene recognition are proposed.

The remainder of this work is organized as follows. Section 2 presents the principles of the SR and recognition algorithm we used. Section 3 presents the experimental process and analyzes the effects of different SR algorithms, SR factors, model library sizes and noise conditions on the recognition rate. Section 4 provides the conclusions.

## 2. Theory

### 2.1. SR Algorithms

#### 2.1.1. Bicubic

The interpolation algorithm estimates unknown pixels on the basis of known pixel values in the neighborhood and image resolution conversion is one of its specific applications. Strictly, the resolution conversion must first reconstruct (or interpolate) discrete data into a continuous curve and then sample at different sampling rates. However, in a real interpolation algorithm, these two steps can be performed in one operation [6]. Bicubic is one of the most common interpolation algorithms. In this algorithm, the value of the function f at the sampling points (x, y) can be obtained by the weighted average of the nearest sixteen sampling points in the grid, which requires two polynomials to cubic interpolation in X and Y directions as shown in Equation (1):

(1) $$f\left(x,y\right)=\sum_{i=0}^{3}\sum_{j=0}^{3}a_{ij}x^{i}y^{j}$$

where $a_{ij}$ is a weighting coefficient, which can be solved by constructing a bicubic basis function. Considering that the Bicubic can perform both up- and down-sampling, all down-sampling processes of images in the experiment in Section 3 use this algorithm. Compared with other SR algorithms, the Bicubic has a smaller calculation amount and has a smoothing effect on the image. However, the actual image is piecewise continuous and the global smoothness assumption is not satisfied in the discontinuous area (e.g., the edge of the image). Thus, when interpolating pixels near the edge of the image, the resulting visual effect will become blurred. Furthermore, the interpolation result is susceptible to noise and pixels with large errors.

#### 2.1.2. MRF

MRF is based on the Markov model and Bayesian theory. The Markov model describes the local statistical dependency relationship between image pixels and considers that a certain pixel value of an image is only related to some pixel values in its neighborhood. Bayesian theory is used to solve the uncertainty problem described by the MRF model, converts the prior knowledge of the image into the prior distribution model and then the maximum posterior probability is used to establish the objective function of the problem to transform the image processing problem into an optimization problem.

J. Diebel and S. Thru [13] used the MRF model for the first time to solve the problem of SR of range images and J. Park et al. [19] made improvements on this basis. The input is defined as the high-res intensity image I and the low-res range image D and the output as the estimated high-res range image $\tilde{D}$. The objective function $E\left(\tilde{D}\right)$ can be constructed as

(2) $$E\left(\tilde{D}\right)=E_{d}\left(\tilde{D}\right)+\lambda_{S}E_{S}\left(\tilde{D}\right)+\lambda_{N}E_{NLS}\left(\tilde{D}\right),$$

where $E_{d}\left(\tilde{D}\right)$ is the data term, $E_{S}\left(\tilde{D}\right)$ is the neighborhood smoothing term and $E_{NLS}\left(\tilde{D}\right)$ is the NLS term. $\lambda_{S}$ and $\lambda_{N}$ are the relative weights to balance the three terms.

Specifically, $E_{d}\left(\tilde{D}\right)$ describes the quadratic distance between $\tilde{D}$ and D:

(3) $$E_{d}\left(\tilde{D}\right)=\sum_{p\in M}{\left[\tilde{D}\left(p\right)-D\left(p\right)\right]}^{2},$$

where p is the index of the pixel position and M is the set of pixels of D.

$E_{S}\left(\tilde{D}\right)$ represents the weighted quadratic distance between pixels in a neighborhood system:

(4) $$\begin{array}{c} E_{S}\left(\tilde{D}\right)=\sum_{p}\sum_{q\in N\left(p\right)}\frac{w_{pq}}{W_{p}}{\left[\tilde{D}\left(p\right)-\tilde{D}\left(q\right)\right]}^{2},\\ W_{p}=\sum_{q}w_{pq},\\ w_{pq}=w_{c}w_{s}w_{e}w_{d} \end{array}$$

where N(p) is the first-order neighborhood of p and $W_{p}$ is the normalization factor. $w_{pq}$ is the confidence weight of pixels p and q, which defines the similarity of neighboring pixels and is the key to introducing the input I into the SR problem. The greater $w_{pq}$ increases the probability that two neighboring pixels have the same range value.$\;w_{pq}$ can be decomposed into four terms on the bases of color similarities $w_{c}$, segmentation $w_{s}$, edge saliency $w_{e}$ and guided bicubic interpolated depth image $w_{d}$.

(1) $w_{c}$ is defined in the YUV color space as
  (5) $$w_{c}=\exp\left\{-\sum_{I\in YUV}\frac{{\left[I\left(p\right)-I\left(q\right)\right]}^{2}}{2\sigma_{I}^{2}}\right\},$$
  where $\sigma_{I}$ controls the relative sensitivity of different color channels.
(2) The image segmentation algorithm in the VLFeat image processing library provided by [20] is used to segment the intensity image into super pixels. For the neighborhood pixels not within the same super pixel, a penalty term is defined as
  (6) $$w_{s}=\{\begin{array}{c} 1\;S_{co}\left(p\right)=S_{co}\left(q\right)\;\\ t_{se}\;otherwise \end{array},$$
  where $S_{co}\left(\cdot \right)$ is the segmentation label and $t_{se}$ is the penalty factor (0 < $t_{se}$ < 1).
(3) $w_{e}$ indicates the weight that depends on the edge saliency response. The edge saliency responses are detected by a set of Gabor filters with different sizes and orientations, which can enhance the smoothness at the edge of the image. The weight is calculated as
  (7) $$w_{e}=\frac{1}{\sqrt{S_{x}{\left(p\right)}^{2}+S_{x}{\left(q\right)}^{2}+1}},$$
  where $S_{x}\left(\cdot \right)$ is the value of the x−axis edge saliency image. If p and q are x−axis neighborhoods, $S_{x}\left(\cdot \right)$ is calculated. If they are y−axis neighborhoods, $S_{y}\left(\cdot \right)$ is calculated.
(4) Allowing the range values to propagate freely with only very sparse data constraints can lead to notable range ambiguity. Therefore, the guide range image $D_{g}$ obtained from the low-res range image through Bicubic is introduced to solve this problem. Similar to the bilateral filter, the weight of the guide range image is defined as
  (8) $$w_{d}=\exp\left\{-\frac{{\left[D_{g}\left(p\right)-D_{g}\left(q\right)\right]}^{2}}{2\sigma_{g}^{2}}\right\}.$$

$E_{NLS}\left(\tilde{D}\right)$ is defined by the anisotropic structural-aware filter [21]:

(9) $$\begin{array}{c} E_{NLS}\left(\tilde{D}\right)=\sum_{p}\sum_{r\in H\left(p\right)}\frac{k_{pr}}{K_{p}}{\left[\tilde{D}\left(p\right)-\tilde{D}\left(r\right)\right]}^{2},\\ K_{p}=\sum_{r}k_{pr}, \end{array}$$

where H(p) is the local window (e.g., 7 × 7) of p and $K_{p}$ is the normalization constant. $k_{pr}$ is the weight of the filter and is defined as

(10) $$\begin{array}{c} k_{pr}=\frac{1}{2}\left\{\exp\left[-{\left(p-r\right)}^{T}\sum_{p}^{-1}\left(p-r\right)\right]+\exp\left[-{\left(p-r\right)}^{T}\sum_{r}^{-1}\left(p-r\right)\right]\right\},\\ \sum_{p}=\frac{1}{\left|H\right|}\sum_{p^{\prime }\in H\left(p\right)}\nabla I\left(p^{\prime }\right)\nabla I{\left(p^{\prime }\right)}^{T},\\ \nabla I\left(p\right)={\left[\nabla_{x}I\left(p\right),\;\nabla_{y}I\left(p\right)\right]}^{T}, \end{array}$$

where ∇I(p) is the x− and y− image gradient vector at pixel p. The structural-aware filter defines the structural similarity of p and r in I.

Finally, the conjugate gradient method is used to solve $\tilde{D}$. The first derivative w.r.t. $\tilde{D}$ on Equation (2) is taken and the derivative equal to zero is set:

(11) $$\frac{∆E\left(\tilde{D}\right)}{∆\tilde{D}}=0.$$

After finishing, the system of linear equations can be obtained:

(12) $$A\tilde{d}=d,$$

where A is a n×n matrix with weight terms and n is the number of pixels in I. $\tilde{d}$ is the desired range values and d is an observed range that is conditionally filled with A. For $\tilde{D}\left(p\right)$, the elements of A and d are filled as

(13) $$A_{pp}=\{\begin{array}{c} 1+\lambda_{S}+\lambda_{N}\;if\;p\in M\\ \lambda_{S}+\lambda_{N}\;otherwise \end{array},$$

(14) $$A_{pq}=-\frac{\lambda_{S}w_{pq}}{W_{p}},$$

(15) $$A_{pr}=-\frac{\lambda_{N}k_{pr}}{K_{p}},$$

(16) $$d_{p}=\{\begin{array}{c} D\left(p\right)\;if.p\in M\\ 0\;otherwise \end{array},$$

where $A_{pp}$ is the element on the diagonal of A, $A_{pq}$ is A’s element at the p-th row and q-th column and $d_{p}$ is the p-th element of d. q∈N(p), r∈H(p) indicate neighborhoods for $E_{S}\left(\tilde{D}\right)$ and $E_{NLS}\left(\tilde{D}\right)$, respectively. Finally, $\tilde{d}$ can be obtained by solving Equation (12) and then $\tilde{D}$ can be reconstructed from $\tilde{d}$.

The specific implementation steps of the MRF algorithm are summarized as follows:

1. Input low-res range image D and high-res intensity image I.
2. Set parameters $\lambda_{S}$, $\lambda_{N}$, $\sigma_{I}$, $\sigma_{g}$ and $t_{se}$.
3. Calculate parameters $w_{pq}$, $W_{p}$, $k_{pr}$ and $K_{p}$ for all pixels according to Equations (4) to (10).
4. Calculate the matrix A and vector d according to Equations (13) to (16).
5. Solve Equation (12) to obtain vector $\tilde{d}$.
6. Reconstruct $\tilde{d}$ to obtain the high-res range image $\tilde{D}$.

#### 2.1.3. JBF

The edge is one of the most important structural features of the image, which reflects the details of the image and belongs to the sensitive high-frequency information of the human eye. Generally, the smoothing effect of the filter will cause blurring in the image edge area, which will reduce people’s ability to distinguish the details of objects. Therefore, in many computer vision applications, filters are required to have edge preservation capabilities.

The bilateral filter is an edge-preserving filter, which has edge-preserving capability whilst smoothing the image and has been widely used in image processing [22]. The kernel function of the bilateral filter can be divided into two parts: the spatial and range filter kernels. Among them, the spatial filter kernel is the same as the Gaussian filter and the range filter kernel is a Gaussian function that calculates the difference between the central pixel and its neighborhood. For point p in image G, the bilateral filtering result can be described as

(17) $$\begin{array}{c} J\left(p\right)=\frac{1}{k_{p}}\sum_{q\in \Omega }w_{pq}G\left(q\right),\\ w_{pq}=\exp\left(-\frac{{\left|p-q\right|}^{2}}{\sigma_{d}}\right)\exp\left(-\frac{{\left|G\left(p\right)-G\left(q\right)\right|}^{2}}{\sigma_{c}}\right), \end{array}$$

where p and q are pixel coordinate., Ω is the filter window, $k_{p}$ is the normalization factor, $w_{pq}$ is the filter kernel and $\sigma_{d}$ and $\sigma_{c}$ are the corresponding weight parameters. Given that the bilateral filter includes a spatial filter and also combines a range filter that describes the intensity similarity, the filter coefficients calculated for the smooth and edge areas of the image are different. The filter coefficient is smaller in the edge area of the image, which can protect the edges better.

J. Kopf et al. [11] first proposed the idea of JBF and applied it to the image SR problem. The principle was to construct the spatial filter from a low-res range image D and the range filter from a high-res intensity image I as shown in Equation (18):

(18) $$\begin{array}{c} \tilde{D}\left(p\right)=\frac{1}{k_{p}}\sum_{q_{↓}\in \Omega }w_{pq}D\left(q_{↓}\right),\\ w_{pq}=\exp\left(-\frac{{\left|p_{↓}-q_{↓}\right|}^{2}}{\sigma_{d}}\right)\exp\left(-\frac{{\left|I\left(p\right)-I\left(q\right)\right|}^{2}}{\sigma_{c}}\right), \end{array}$$

where $\tilde{D}$ is the high-res range image to be restored and $p_{↓}$ and $q_{↓}$ denote (possibly fractional) coordinates of pixels in D corresponding to p and q in $\tilde{D}$.

#### 2.1.4. SGJF

The filter of the JBF algorithm only considers the spatial Gaussian kernel and the intensity Gaussian kernel; that is, it assumes that where the intensity image gradient is large, the range image gradient is also large. However, the range image actually often makes smooth changes in sections; that is, the range information belonging to the same area of the object is constant or gradual, whereas the corresponding color guide image may have a certain texture structure that will cause the latter to be introduced into the super-resolved images after filtering. This phenomenon is called ‘texture transfer’.

To reduce the effect of intensity image texture on the super-resolved images, we propose the SGJF algorithm. This algorithm adds the range information of the range image as a coefficient of the filter and reduces the influence of the intensity image texture by strengthening its weight. The specific method is to interpolate the low-res range image (e.g., using the Bicubic algorithm) to obtain a high-res range guided image $R_{g}$ and then express it in a form of similar intensity as a Gaussian filter kernel as

(19) $$\exp\left(-\frac{{\left|R_{g}\left(p\right)-R_{g}\left(q\right)\right|}^{2}}{\sigma_{u}}\right),$$

where $\sigma_{u}$ is the weight parameter; a larger value increases the weight. Finally, Equation (19) is added as a coefficient to Equation (18) to obtain the final filter kernel as

(20) $$w_{pq}=\exp\left(-\frac{{\left|p_{↓}-q_{↓}\right|}^{2}}{\sigma_{d}}\right)\exp\left(-\frac{{\left|I\left(p\right)-I\left(q\right)\right|}^{2}}{\sigma_{c}}\right)\exp\left(-\frac{{\left|R_{g}\left(p\right)-R_{g}\left(q\right)\right|}^{2}}{\sigma_{u}}\right),$$

The specific implementation steps of the SGJF algorithm are summarized as follows:

1. Input low-res range image D and high-res intensity image I.
2. Calculate the magnification according to the sizes of I and D and then calculate the guide image $R_{g}$ using the Bicubic.
3. Set the filter window size ω and weight parameters $\sigma_{d}$, $\sigma_{c}$ and $\sigma_{u}$.
4. Calculate the filter kernel $w_{pq}$ of pixel p according to Equation (20).
5. Calculate the range value $\tilde{D}\left(p\right)$ at pixel p according to Equation (18).
6. Repeat steps 4 and 5 for all pixels to obtain a high-res range image $\tilde{D}$.

**Figure 1:**
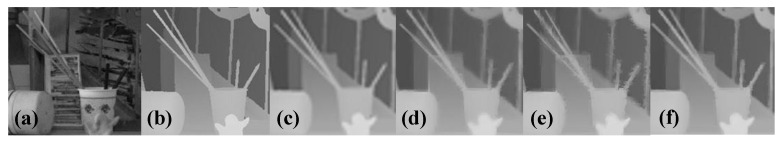
Visual comparison of super-resolved images of the Middlebury Art dataset. (**a**) Original high-res intensity image. (**b**) Original high-res range image. Super-resolved image by (**c**) Bicubic, (**d**) Markov random field (MRF), (**e**) joint bilateral filter (JBF) and (**f**) self-guided joint filtering (SGJF).

Figure 1 shows the SR results of the Middlebury Art dataset with an SR factor of 4. The edges of the super-resolved image obtained by Bicubic are blurred. The super-resolved image obtained by MRF is less affected by the texture of the intensity image because the global optimization algorithm averages the local error to the entire image. However, it has fuzzy restoration details. The super-resolved image obtained by JBF is affected by the intensity image and blurring occurs at the slender edges caused by ‘texture transfer’. The super-resolved image obtained by SGJF has clear image edges and is less affected by the intensity image texture.

### 2.2. Recognition Algorithm

Target recognition based on ladar range images generally includes two processes of model acquisition and scene recognition and four steps of noise suppression, image segmentation, feature description and feature classification [23]. The model refers to the pre-defined 3D information of the target object to be recognized (i.e., the range images with known classification results) and the scene refers to the measurement data of the ladar (i.e., the range images to be classified). Generally, the range images acquired by ladar always contain various noises, which seriously affect the accuracy of the recognition algorithm. Therefore, the first step in target recognition is to suppress noise. Recognition algorithms based on local features typically require no image segmentation, whereas the recognition algorithms based on global features must undergo image segmentation. Feature description is intended to describe the geometric features of the target object in 3D space through mathematical language. Moreover, the feature classification refers to the classification of the geometric features described by mathematical language through algorithms to finally achieve target recognition.

#### 2.2.1. Pre-Processing

Pre-processing includes noise suppression and image segmentation. Since the acquired range images are prone to produce some holes or obvious noises at the edges and areas with low reflectance of the target object, it is necessary to fill in these missing areas and eliminate outliers before processing the image. We used a statistical-based method [24] to complete the filling process by replacing the zero and extreme pixels with the statistical mode of the surrounding 25 pixels. This method can return sharper edges than using statistical mean values. Notably, we only corrected the outliers and did not change other known pixels, thereby avoiding blurring of the image edges.

After eliminating the outliers, we removed the ground and partial noise by fitting the plane. The least square method was initially used to fit the plane function of the ground through the outermost ring range data of the range image. The ground and partial noise could then be removed by giving up the range data of the plane and its vicinity. After that, a binary operation was used to extract the region of the target object to complete the image segmentation. However, when there was strong noise in the range image, the above operation was not enough to remove all the noise. It was also necessary to clean up the remaining noise spots by the method of extraction of connected components to obtain a clean binary image.

To improve the robustness, we acquired three range images for each pose of the target object and pre-processed them separately. The final binary image was obtained by voting after accumulating the three pre-processed images.

#### 2.2.2. Combined Moment Invariants

Moment invariant as a global feature description vector has important applications in the field of ladar range image target recognition [25]. In general, lower order moments can reflect the overall characteristics of the image and higher order moments can reflect the detailed characteristics of the image. We used the combined moment invariants composed of Hu’s seven moment invariants [26] constructed by the second- and third-order central moments and three of the affine moment invariants [27] constructed from algebraic invariant theory as the feature descriptors. The combined moment invariants have translation, rotation and scale invariance and are written as

(21) $$\phi_{1}=\eta_{20}+\eta_{02},$$

(22) $$\phi_{2}={\left(\eta_{20}-\eta_{02}\right)}^{2}+4\eta_{11}^{2},$$

(23) $$\phi_{3}={\left(\eta_{30}-3\eta_{12}\right)}^{2}+{\left(3\eta_{21}-\eta_{03}\right)}^{2},$$

(24) $$\phi_{4}={\left(\eta_{30}+\eta_{12}\right)}^{2}+{\left(\eta_{21}+\eta_{03}\right)}^{2}$$

(25) $$\phi_{5}=\left(\eta_{30}-3\eta_{12}\right)\left(\eta_{30}+\eta_{12}\right)\left[{\left(\eta_{30}+\eta_{12}\right)}^{2}-3{\left(\eta_{21}+\eta_{03}\right)}^{2}\right]+\left(3\eta_{21}-\eta_{03}\right)\left(\eta_{21}+\eta_{03}\right)\times \left[3{\left(\eta_{30}+\eta_{12}\right)}^{2}-{\left(\eta_{21}+\eta_{03}\right)}^{2}\right],$$

(26) $$\phi_{6}=\left(\eta_{20}-\eta_{02}\right)\left[3{\left(\eta_{30}+\eta_{12}\right)}^{2}-{\left(\eta_{21}+\eta_{03}\right)}^{2}\right]+4\eta_{11}\left(\eta_{30}+\eta_{12}\right)\left(\eta_{21}+\eta_{03}\right),$$

(27) $$\phi_{7}=\left(3\eta_{21}-\eta_{03}\right)\left(\eta_{30}+\eta_{12}\right)\left[{\left(\eta_{30}+\eta_{12}\right)}^{2}-3{\left(\eta_{21}+\eta_{03}\right)}^{2}\right]+\left(3\eta_{21}-\eta_{03}\right)\left(\eta_{21}+\eta_{03}\right)\times \left[{\left(\eta_{30}+\eta_{12}\right)}^{2}-{\left(\eta_{21}+\eta_{03}\right)}^{2}\right],$$

(28) $$\phi_{8}=\frac{1}{\eta_{00}^{4}}\left(\eta_{20}\eta_{02}-\eta_{11}^{2}\right),$$

(29) $$\phi_{9}=\frac{1}{\eta_{00}^{10}}\left(\eta_{30}^{2}\eta_{03}^{2}-6\eta_{30}\eta_{12}\eta_{21}\eta_{03}+4\eta_{30}\eta_{12}^{3}+4\eta_{03}\eta_{21}^{3}-3\eta_{21}^{2}\eta_{12}^{2}\right),$$

(30) $$\phi_{10}=\frac{1}{\eta_{00}^{7}}\left(\eta_{20}\eta_{21}\eta_{03}-\eta_{20}\eta_{12}^{2}-\eta_{11}\eta_{30}\eta_{03}+\eta_{11}\eta_{21}\eta_{12}+\eta_{02}\eta_{30}\eta_{12}-\eta_{02}\eta_{21}^{2}\right),$$

where $\eta_{pq}$ is the normalised center moment. To adjust the distribution range of the ten feature description vectors, we represented them in logarithm as

(31) $$\mu_{i}=\left|\log\left|\phi_{i}\right|\right|,\;i=1,\;2,\;\cdots .$$

#### 2.2.3. BPNN

All of the features of all of the models described by combined moment invariants were then used to train the classifier for scene recognition. BPNN as a classifier is currently widely used to classify ladar range image features [28]. It uses gradient descent to calculate the correction amount of network connection weights and continuously corrects network weights from back to front until the network output error reaches a predetermined threshold.

Generally, BPNN includes at least three layers: an input layer, a hidden layer and an output layer. The input layer to the output layer is one-way propagation and the relationship between the input data and the output result is non-linear. The three-layer BPNN used in the experiment can be expressed as [29]

(32) $$g_{k}\left(X\right)\equiv z_{k}=f\left[\sum_{j=1}^{n_{H}}w_{jk}f\left(\sum_{i=1}^{d}w_{ji}x_{i}+w_{j0}\right)+w_{k0}\right],$$

where f is the transfer function, x is the input vector, z is the output vector, w  is the weight of each layer and d and $n_{H}$ denote the dimensions of the input vector and the hidden layer, respectively.

## 3. Experiments and Results

This section is divided into four parts. Section 3.1 introduces the overall steps of the experiments and the platform used to acquire range images. Section 3.2, Section 3.3 and Section 3.4 are three independent experiments and each experiment is described in the order of experiment purpose, organization method and result analysis.

### 3.1. Experimental Steps and Platform

#### 3.1.1. Experimental Steps

**Figure 2:**
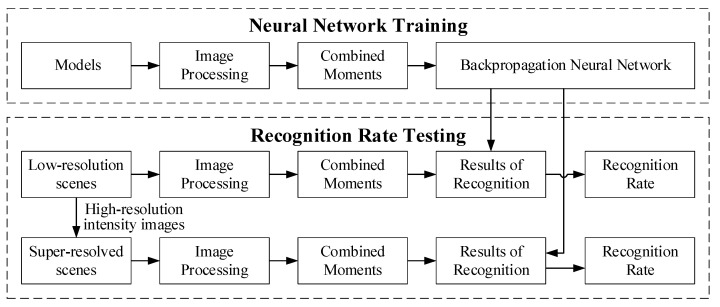
Block diagram for the super-resolution (SR) recognition system.

Figure 2 illustrates the operating steps of the designed SR recognition system for range images, which is divided into neural network training and recognition rate testing. For training neural networks, the noise of models was initially removed through image pre-processing and converted into binary images with target shape information. The combined moments were then used as the feature descriptor to calculate the feature vectors of the binary image. Finally, the BPNN was trained by these feature vectors as the feature classifier for scene recognition. In the process of recognition rate testing, the low-res scenes and their super-resolved images were calculated in the same way to obtain feature vectors. These were then inputted into the trained BPNN to obtain the classification results before calculating the recognition rate. Finally, the effect of the SR algorithms on recognition was evaluated by comparing the two sets of recognition rates.

#### 3.1.2. Image Acquisition Platform

**Figure 3:**
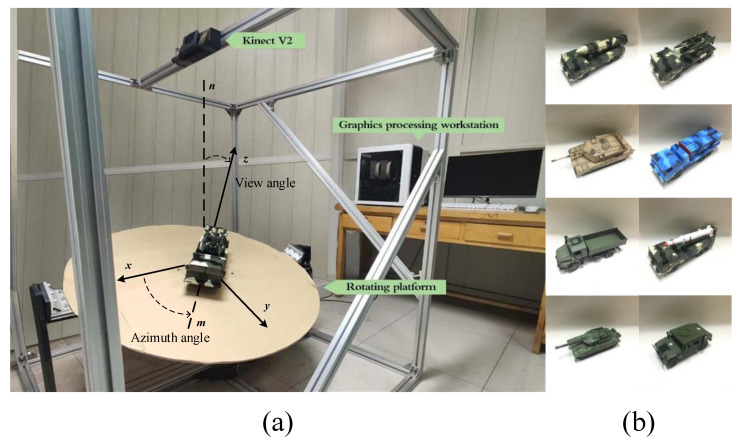
Experimental setup. (**a**) Image acquisition platform. (**b**) Target objects.

**Figure 4:**
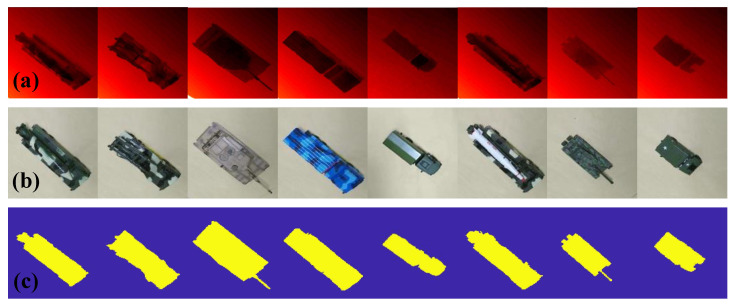
Acquired and pre-processed target object images. (**a**) Range images. (**b**) Intensity images. (**c**) Binary images.

An image acquisition platform (Figure 3a) was designed to acquire range and intensity images of eight target objects (Figure 3b). This platform was equipped with a Kinect V2, which combined a Time-of-Flight (ToF) camera and an RGB camera. Moreover, it acquired range and intensity images of the target objects at any attitude angle (azimuth angle 0°–360° and view angle 0°–80°). The acquired images were divided into training and test sample sets in which the former was used to simulate the model library to train the BPNN and the latter was used to simulate the scene library to test the recognition rate. We defined the acquired range image, with a spatial resolution of 320 × 320 pixels, as the original high-res range image (OHRRI) and the intensity image aligned with the OHRRI as the original high-res intensity image (OHRII) in the experiments. In addition, given that temperature could have a profound influence on the credibility of the range data acquired by the Kinect V2 ToF camera, all data of the experiment were acquired after the Kinect V2 was run for 30 min to avoid temperature effects [30]. Figure 4 shows the range and intensity images of the eight target objects and the binary image obtained after pre-processing.

Table 1 shows the parameters of the SR algorithms. For an objective comparison, the values of the common parameters of JBF and SGJF were set to be the same and the value of ω was different according to the SR factor as s. Bicubic used the function imresize () in MATLAB. For BPNN, the input layer node was set to 10 to correspond to 10 moment invariant feature description vectors and the output node was set to 8 to correspond to 8 target objects. The transfer function of its first layer was the ‘Tansig’ function and the second layer was the ‘Purelin’ function. The number of iterations was set to 500. Considering that different training sample sets contained different numbers of training samples, the number of hidden layer neurons was also different when training BPNN to avoid underfitting or overfitting. The selection of all parameters represented the best result of multiple tests.

### 3.2. Arbitrary Attitude Angle Recognition

In the case where the SR algorithm was not used, the recognition effect of the recognition system on scenes with any attitude angle within the acquisition range of the platform was tested and the influence of the model library size on the recognition rate was analyzed. A test sample set for simulating a scene library was firstly established. At 10° intervals, range images of each target object at view angles of 5° to 75° and azimuth angles of 5° to 175° were acquired; thus, the test sample set contained 1152 range images. Next, training sample sets for simulating model libraries were established. Still at 10° intervals, range images of each target object at view angles of 10° to 80° and azimuth angles of 0° to 180° were acquired and one image when the view angle was 0°; thus, a total of 153 range images of each target object were acquired. Different numbers of range images were finally extracted as training samples to establish the training sample sets with different sizes. According to the view angle, the number of range images taken at different intervals were as follows: (1) three at 40°, (2) five at 20° and (3) nine at 10°. For azimuth, the numbers of range images taken at different intervals were as follows: (1) three at 90°, (2) four at 60°, (3) seven at 30°, (4) 10 at 20° and (5) 19 at 10°. Combining the view and azimuth angles, 15 training sample sets with different sizes were obtained. Table 2 shows the recognition rates of the test sample set by 15 BPNNs trained by the training sample sets and the number of samples contained in each training sample set is shown in parentheses.

From the table, the recognition rate of the test sample set by the BPNN was typically positively related to the training sample set size. However, when the training sample set had fewer samples selected at the view angle (or azimuth angle), the recognition rate was low even if many samples were selected in another direction. By contrast, when the training sample set size increased to a certain number, the recognition rate grew slowly or even stopped. If we wanted to maintain the recognition rate above 95%, a minimum of 232 training samples were required and if we wanted to maintain the recognition rate above 90%, a minimum of 104 training samples were required.

### 3.3. SR Recognition of Scene-Model with the Same Resolution

#### 3.3.1. Experiment without Noise

**Figure 5:**
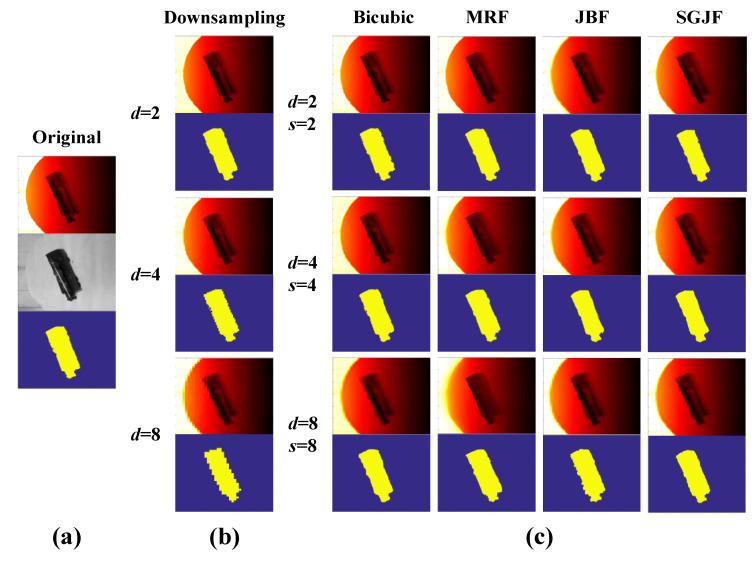
Explanation of the experiment phenomenon. (**a**) Original images. (**b**) Down-sampled images. (**c**) Super-resolved images.

The SR recognition of low-res scenes without noise was tested and the resolution of their super-resolved scene was the same as the resolution of the model. The down-sampling factor was defined as d and the SR factor as s. Firstly, OHRRIs and OHRIIs of each target object (Figure 5a) were acquired. OHRRIs were then down-sampled by factors d of 2, 4 and 8 to simulate low-res scenes (Figure 5b). Finally, the four SR algorithms described above were applied to low-res scenes and s was the same as d (Figure 5c). OHRIIs were used as guidance for SR algorithms requiring intensity image guidance.

The binary image could well represent the contour of the target object and the effect of the down-sampled images slowly worsened as the factor d increased. For super-resolved images, the difference was not very obvious when the factor s was small. When the factor was large, the edge of the Bicubic became blurred, MRF was difficult to see the details, JBF had obvious texture transfer and SGJF was less affected by the texture of intensity and the details were clear.

**Figure 6:**
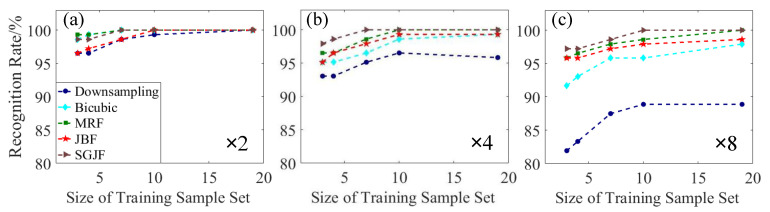
Comparison of recognition rates of different SR algorithms and factors without noise. (**a**) d=s=2. (**b**) d=s=4. (**c**) d=s=8.

To test the recognition rate, the training sample sets were established by OHRRIs and the test sample sets were established by down-sampled images and their SR images. Compared with Section 3.2, the attitude angle of the acquired image was simplified to only acquire images with a view angle of 40°, whereas the acquisition method of the azimuth angle was unchanged. For the training sample set, extracting the azimuth angle in the same way as in Section 3.2 established five training sample sets with different sizes and the number of range images they contained was 24, 32, 56, 80 and 152, respectively. For the test sample set, each target object first acquired 18 images. These were then down-sampled by factors d of 2, 4 and 8 to establish three down-sampling test sample sets. Next, the four SR algorithms were applied to the three down-sampling test sample sets with the same s as d. According to the different SR algorithms and factors s, 12 SR test sample sets were established. Therefore, a total of 15 test sample sets were finally established and each of them contained 144 (18 × 8) images. Figure 6 shows the recognition results of the 15 test sample sets by five BPNNs trained by the training sample sets.

Figure 6 shows that the recognition rate of all SR test sample sets was higher than that of the down-sampling test sample sets, especially when the factor was large. It represented that the SR algorithm helped improve the recognition rate of the acquired low-res scenes; a high factor increased the improvement. However, for each SR algorithm, the recognition rate of the large factor sample set was lower than that of the small factor sample set. Specifically, the SGJF test sample set often maintained a high recognition rate. The JBF test sample set had a lower recognition rate than others when the factor was small. However, as the factor increased, its decrease was smaller. The MRF test sample set had the highest recognition rate when the factor was small and was only lower than the SGJF test sample set when the factor was large. The recognition rate of the Bicubic test sample set decreased the most with the increase in the factor.

#### 3.3.2. Experiment with Noise

**Figure 7:**
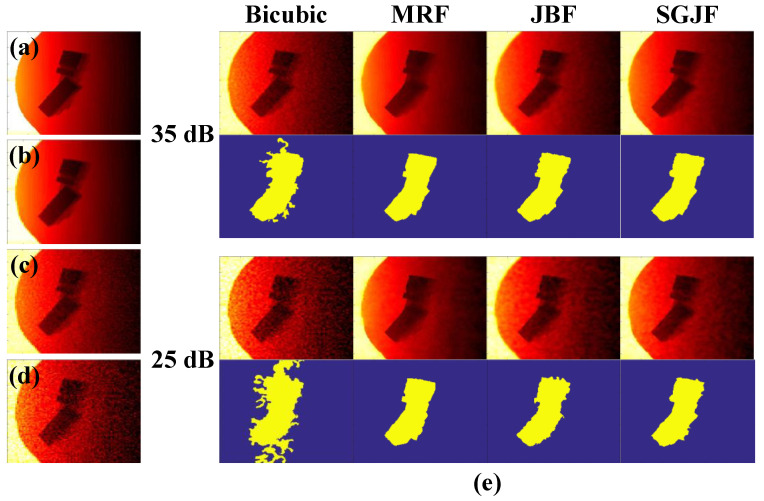
Explanation of the experiment phenomenon. (**a**) Original high-res range image (OHRRI). (**b**) Down-sampled image at d=4. (**b**,**c**) with 35 dB noise. (**b**,**d**) with 25 dB noise. (**e**) Super-resolved images of (**c**,**d**) and their binary images.

In practical applications, the ladar range image often contains various noises, mainly Gaussian noise. To test the influence of noise on the SR algorithm in the system, this section used the same experimental procedure as Section 3.3.1. The difference was that before running the SR algorithm, Gaussian noise with a signal-to-noise ratio (SNR) of 35 dB and 25 dB was first added to the down-sampled image. Figure 7 shows the super-resolved images and their binary images when the factor was 4. The Bicubic algorithm was incapable of suppressing noise and the noise seriously affected the extraction of binary images. The other three algorithms obviously suppressed the noise, where the MRF super-resolved range image was the clearest and its binary image obtained smooth edges even when the noise intensity was large. Both JBF and SGJF super-resolved range images were somewhat affected when the noise intensity was large. In addition, the JBF super-resolved range image was also affected by the intensity image texture. These findings were all reflected in the binary image.

**Figure 8:**
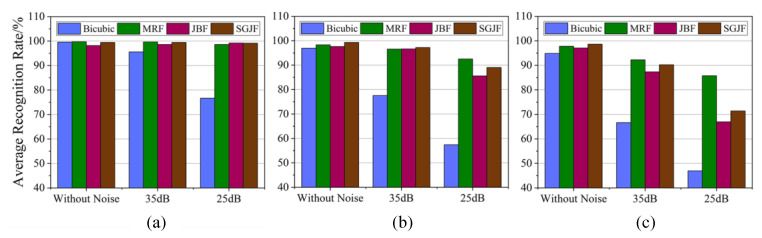
Comparison of the average recognition rates of different SR algorithms and factors with noise. (**a**) d=s=2. (**b**) d=s=4. (**c**) d=s=8.

The five BPNNs trained by the training sample set in Section 3.3.1 were still used to test the recognition rate and the acquisition of the test sample set also followed the steps in Section 3.3.1, except for adding noise with a different SNR. According to the result of Section 3.3.1, the recognition rate increased with the training sample set size and the recognition rate curves of different SR algorithms basically did not cross. Therefore, the average recognition rate (i.e., the average of the recognition rates of five different BPNNs) was used to describe the recognition results of the system under noisy conditions, as shown in Figure 8.

Overall, a larger factor hastened the decrease in the average recognition rate and 25 dB decreased faster than 35 dB. Specifically, the Bicubic algorithm was extremely sensitive to noise and was unsuitable for use when the noise intensity and factor was large. The MRF algorithm performed best in noise and the recognition rate of its test sample set only decreased slowly with the increase in noise intensity and factor. However, it needed to adjust more parameters and the calculation was more complicated. The JBF and SGJF algorithms were also robust to noise. When the factor or noise intensity was low, they were less affected. However, when the factor and noise intensity increased simultaneously, the average recognition rate dropped significantly and the gap between the JBF and SGJF algorithms also increased. Taken together, the average recognition rate of the SGJF algorithm was significantly smaller than the MRF algorithm only when the noise intensity and factor were at the maximum, whilst it was superior or similar to other algorithms in other cases. Therefore, the SGJF algorithm had a very good comprehensive recognition performance.

### 3.4. SR Recognition of Scene-Model with Multiple Resolutions

**Figure 9:**
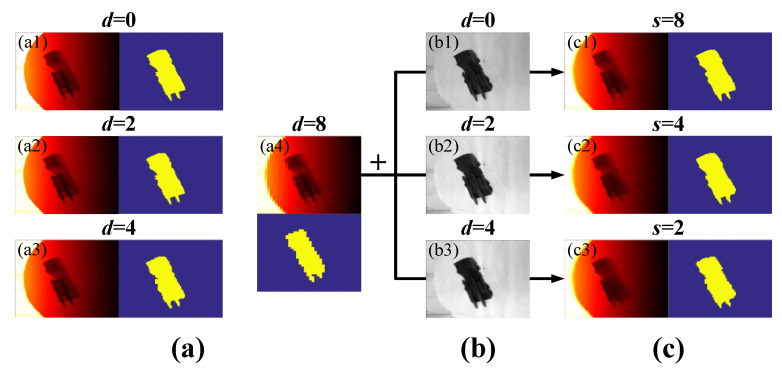
Explanation of the experiment phenomenon. (**a**) OHRRI and its down-sampled images. (**b**) OHRII and its down-sampled images. (**c**) Super-resolved images of the original low-res range image (OLRRI).

Recognizing multi-resolution (multi-res) super-resolved scene libraries by multi-res model libraries without noise was tested where the resolution of the super-resolved scene library may have been lower than, equal to or higher than the model library. Firstly, OHRRIs and OHRIIs of each target object (Figure 9(a1,b1)) were acquired. OHRRIs were then down-sampled by factors d of 2, 4 and 8 (Figure 9(a2–a4)) and the down-sampled image with d=8 was called the original low-res range image (OLRRI). Meanwhile, the down-sampled images of OHRIIs with factors d as 2 and 4 (Figure 9(b2,b3)) together with OHRIIs were used as the guide images. Finally, the SGJF algorithm with a very good comprehensive recognition performance was applied to OLRRIs and the factors s were 2, 4 and 8 (Figure 9(c1–c3)). The smaller the factor d of the down-sampled image and the larger the factor s of the SR image, the clearer the image and the smoother the edges of their binary image.

**Figure 10:**
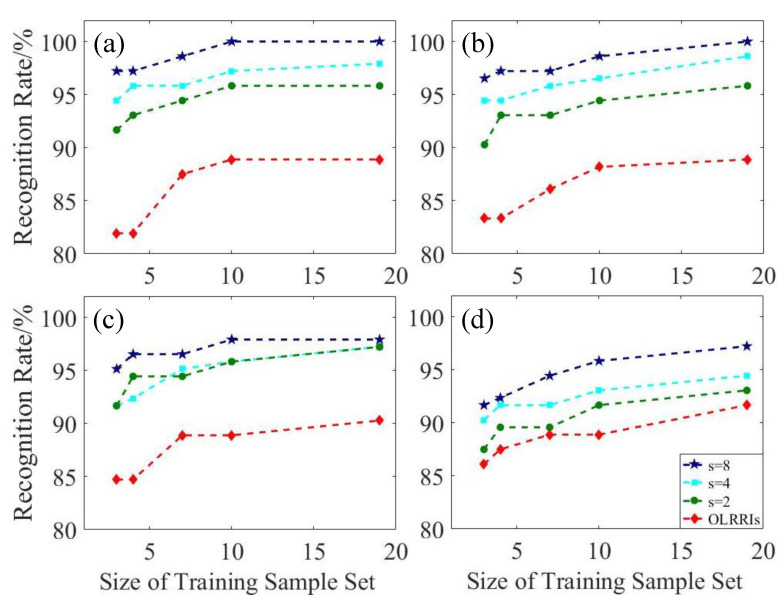
Comparison of the recognition rate of the multi-res training sample set with the multi-res test sample set without noise. The resolution of the training sample set was (**a**) OHRRI. (**b**) Down-sampled image of OHRRI by factor d of 2. (**c**) Down-sampled image of OHRRI by factor d of 4. (**d**) Down-sampled image of OHRRI by factor d of 8 (i.e., OLRRI).

To test the recognition rate, training and test sample sets were established and the attitude angle of the image in each sample set was the same as Section 3.3.1. The images in the training sample sets contained four different resolutions, which were OHRRI and its down-sampled image with factors d of 2, 4 and 8 (i.e., OLRRI). Moreover, each resolution image established five training sample sets with different sizes. Therefore, a total of 20 training sample sets were obtained. In addition, the resolutions of the four test sample sets established were also different. They were composed of OLRRIs and their super-resolved images obtained by SGJF algorithm with factors s of 2, 4 and 8. Figure 10 shows the recognition results of the four test sample sets by 20 BPNNs trained by the training sample sets.

Regardless of which resolution training sample set was used to train the BPNN, the higher the resolution of the test sample set (i.e., the greater s), the higher the recognition rate. This finding was explained by the fact that the difference between different test samples increased as the resolution increased. Therefore, test samples with a higher resolution were easier to distinguish and recognize. Conversely, the results of each test sample set recognized by training sample sets with different resolutions were compared longitudinally. When the resolutions of the test and training sample sets were the same, the recognition rate was higher than when their resolutions were different.

According to the experimental results, the method of using the SR algorithm in actual scene recognition is obtained as follows. (1) When a low-res scene is acquired, if it can be super-resolved then it should be super-resolved. (2) If multiple SR factors are available to choose from, the largest one should be selected even if the resolution of the super-resolved scene is higher than that of the model in the model library. (3) If only one SR factor exists to choose from, but multiple model libraries are available with different resolutions, the model library with the same resolution as the super-resolved scene should be selected.

## 4. Conclusions

This work proposed an SR algorithm for range images based on SGJF, which can reduce the effect of intensity image texture on super-resolved images by adding the range information of the range image as a coefficient of the filter. We also constructed an SR recognition system for range images, which acquired range and intensity images of eight military target objects through the image acquisition platform to establish training and test sample sets for the simulation model and scene libraries and used the Bicubic, JBF, MRF and SGJF algorithms to test the effect of the SR algorithm on the recognition of ladar range images. The recognition of scenes with arbitrary attitude angles by model libraries of different sizes was initially tested. The results demonstrated that the recognition rate was positively correlated with the model library size. However, the recognition rate grew slowly or even stopped when it increased to a certain number. The recognition of the system under different noise conditions was then tested when the resolutions of the super-resolved scene and the model were the same. The results demonstrated that all the SR algorithms we tested can help improve the recognition rate of low-res scenes; moreover, a larger factor increases the improvement. The SGJF algorithm we proposed largely avoids the problem of ‘texture transfer’. It had the highest recognition rate when no noise occurred and showed noise robustness after adding noise. Therefore, the SGJF algorithm has a very good comprehensive recognition performance. Finally, the recognition of multi-res super-resolved scene libraries by multi-res model libraries was tested. The results demonstrated that regardless of the resolution of the model library, an increase in the resolution of the super-resolved scene also increased the recognition rate. In addition, using the model library with the same resolution as the super-resolved scene could obtain the highest recognition rate. According to the experimental results, suggestions on the use of SR algorithms in actual scene recognition are proposed. Our future work includes studying the application of the SR algorithm in the recognition of radar range images based on local features.

## Figures and Tables

**Table 1:** SR algorithm parameters.

MRF	Value	JBF	SGJF	Value
$\lambda_{S}$	0.20	$\sigma_{d}$		3
$\lambda_{N}$	0.20	$\sigma_{c}$		0.10
$\sigma_{I}$	0.05		$\sigma_{u}$	0.15
$\sigma_{g}$	0.05	ω		3 (s = 2)
$t_{se}$	0.70			5 (s = 4)
				9 (s = 8)

**Table 2:** Recognition rates on different training sample sets.

	Azimuth Interval	90° (3)	60° (4)	30° (7)	20° (10)	10° (19)
View Interval						
**40° (3)**		87.50% (56)	87.76% (72)	89.41% (120)	92.18% (168)	93.40% (312)
**20° (5)**		92.80% (104)	94.36% (136)	95.05% (232)	95.66% (328)	96.18% (616)
**10° (9)**		94.79% (200)	96.18% (264)	96.88% (456)	97.22% (648)	97.22% (1224)
